# Menorrhagia

**Published:** 1854-11

**Authors:** Edward Rigby

**Affiliations:** Senior Physician to the General Lying-in Hospital; Examiner in Midwifery at the University of London


*Menorrhagia.* By EDWARD RIGBY, M.D., etc., Senior Physician to the General Lying-in Hospital; Examiner in Midwifery at the University of London.—In selecting cases of menorrhagia coming on at that period of a woman's life when the menses are either about to cease, or ought to have done so, and attended with local symptoms and conditions of the uterus which I have considered to be of a suspicious, if not dangerous, character, let it be understood, that I by no means point them out as cases of actual malignant disease, but merely as exhibiting more or less of a disposition thereto, although still, in many instances, admitting of considerable relief by treatment.

If we examine into the history of malignant uterine disease, we shall, in most instances, find that there has been an early passive stage of considerable duration; that the health has become gradually impaired, the uterine functions deranged; in women who have been pregnant several times, their two or three last pregnancies have probably terminated in abortion, which has come on without any very assignable cause. I am aware that this applies equally well to non-malignant disease, like fibrous tumor; but it is not the less worthy of note, as it often furnishes us with the earliest data respecting the commencement of the disease, and enables us, in reviewing the history of the case, to trace it back to an earlier period than would otherwise have been suspected. That abortion is not an invariable forerunner of malignant or non-malignant disease, is proved by the well-known fact of pregnancy being associated with every form of it, and running its course undisturbed to a late, or even the full period, even under circumstances where it could scarcely have been supposed possible. This, however, must be rather looked upon as an exception to the rule, that pregnancy occurring during the two or three years which precede the outbreak of uterine disease mostly terminates in abortion. I hold, therefore, that abortion coming on without any very evident cause in a woman past 40, is a suspicious symptom, and the more worthy of attention, as the early symptoms of uterine disease are generally too inappreciable to attract even the notice of the patient, and are of a nature about which she would not willingly consult a Medical man if she could possibly avoid it.

In most instances, abortion is rather a retrospective symptom, to afford some probable clue as to how long the disease has existed, when the fears of the patient and suspicions of the Medical man have been excited by attacks of menorrhagia. It is to these attacks (of menorrhagia) in their earliest stages and slightest degrees, and more particularly to that group of symptoms which I mentioned in my last report as chiefly connected with chylo-poietic derangement, and to the anæmic, chlorotic appearance of the patient, that I am peculiarly anxious to draw the attention of the Profession, because this is the period when treatment will be of most avail, and produce the most striking effects.

Mrs. H., aged 50; married 21 years; four children; aborted in her last three pregnancies; short and stout; pale and flabby.

Dec. 10, 1849.—Profuse bloody discharge, coming on irregularly, sometimes bright, at others dark. The periods have varied a good deal of late, sometimes appearing every fortnight, at others only once in five weeks. They last more than a week, and are profuse for the first four or five days; sometimes preceded for some hours by rather sharp pain about the hips, which she is in the habit of relieving by a sedative pill. These attacks are attended with much nervous depression. Has pain sometimes of one and sometimes of the other groin. Tongue clean, but sulcated. Bowels regular.

The catamenia have been gradually becoming profuse during the last ten years, and first showed this disposition two years after her last aborion. During the last year she has had a slight watery discharge, which ttiffens her linen.

*Examination per vaginam.*—Os uteri high up, small and soft; cervix short; uterus much enlarged. The uterine sound would not pass at first beyond one inch, but the dilator passed easily through the os internum, and then the sound passed 3½ inches. A quantity of thick, clotty, dark brown fluid came away. The fundus is inclined to the right side.

℞ Pil. hydrarg. gr. iij., ferri sulph. gr. ij., extr. hyoscyami gr. v. M. ft. pil. ij. o. n. s. Mist. potassæ bicarb: et nitratis ter die.

12th —Catamenia coming on. Omitt. mist.

℞ Acidi gallici, extr. hyoscyami aa. gr. v. M. ft. pil. ij., bis die sumend.

15th.—Catamenia came on freely, but not profusely, and without clots. The discharge was brown and rather thick. Has not felt weakened by it. Bowels confined.

℞ Pil. hydrarg. extr. coloc. co., extr. hyosc. aa. ℈j. M. ft. pil. xij., sumat ij. h. s.

20th.—Was seized during the night with severe pain of spasmodic character in different parts of the abdomen, which feels full and loaded; she bears gentle pressure, but vomits occasionally; pulse feeble; looks very ill.

℞ Ol. ricini ʒvj. statim; et repet. si opus sit. Enema magnum.

℞ Hydrarg. c. cretâ, pulv. ipecac. comp. aa. gr. v., h. ss.

24th.—Is much better; has continued the pills every night, and castor-oil every morning; large quantities of knotty scybala have come away daily with very great relief; still, however, the abdomen is large and doughy.

℞ Ext. hyosc., ext. gentianæ aa. gr. v., o. n.

℞ Ferri sulph. gr. xvj.; magnesiæ sulph. ℥j.; acidi sulph. dil. ʒj; syrupi rhœados ℥ss.; aquæ menthæ pip. ℥vijss. M. ft. mistura, sumat cochl. magn. ij. primo mane.

1852, Dec. 2.—Has had no catamenia or discharge of any kind for a year; is looking better; has kept the bowels regular with a rhubarb draught and an occasional blue pill. Has pains (apparently hepatic) across the middle of the back and right shoulder; lies best on the right side; these pains come on every four or five weeks, at which time the right hypochondrium is tender.

℞ Pil. hydr. ext. hyosc. aa. gr. v. h. s. p. r. n.

℞ Acidi hydrochlor. dil., acidi nitrici dil. aa. ʒj.; liq. tarax. ℥j.; infusi aurantii comp. ℥vij. M. ft. mistura cujus sumat cochl. magna ij., ter die.

℞ Sodæ potassio tart. mannæ opt. aa. ℥ss.; pulv. rhœi ʒss.; aq. menthæ pip. ℥iij. M. ft. haust. sumat demid. primo mane post pilulas.

1854, April 1.—Is suffering from pains of a spasmodic character, commencing beneath the right scapula, and extending round to the left side; evacuations dark; urine rather scanty; no discharge of any kind; the right hypochondrium is tender during an attack. Has taken pil. hydr. three times during the week. Rep. pil. hydr. p. r. n.

℞ Liq. taraxaci cochl. min. j. om. nocte ex aquâ vel lacte.

℞ Lin. camph. co. ℥iiss.; tinci. opii ℥ss. M. ft. linimentum parti dolenti applicand.

In this case, it is worthy of the remark, that her three last pregnancies terminated prematurely in abortion, that the catamenia, in two years after the last abortion, had gradually assumed a menorrhagic character, and latterly had become irregular as to the periods, and variable as to the appearance of the discharge; moreover, during the last year, she had had in the intervals a watery discharge, which rendered the linen stiff on becoming dry. The uterus was larger and more bulky than natural; the os uteri internum did not admit the sound; but, as is frequently seen with the urethra, allowed a larger blunt instrument to pass; the uterine cavity was increased in size, and filled with dark-brown, grumous fluid. The sum of this evidence is decidedly of an unfavorable character; it shows that the uterus was much enlarged from passive congestion, and that the upper part of the canal of the cervix was sufficiently obstructed by swelling to retain a quantity of the last catamenial discharge.

The bowels were stated to be regular; I did not, therefore, venture to give a purgative in her exhausted state, and even combined the small dose of blue pill with a tonic, and as soon as there were evidences that the catamenia were returning, I gave her the gallic acid with good effect; the discharge, although free, did not become profuse, and she was spared the severe prostration which she had suffered on previous occasions; further observation, however, convinced me that the bowels were much loaded; purgative medicine of mild but effective character was given, and its action still further assisted by a large enema; great quantities of scybala were dislodged with striking relief, and their evacuation was followed by general improvement of her symptoms. There can be little doubt but that this intestinal accumulation had tended much to aggravate the uterine congestion, and thereby the hæmorrhage; and, *vice versâ*, that the enlarged uterus, thus pressed upon by the loaded bowels, had much increased the constipation by the pressure which it exerted upon the rectum.

I regret much that I had not the opportunity of making another examination, that I might ascertain what amount of change had taken place in the uterus, and especially how far it had diminished in size; but her improved state of health rendered it unnecessary.—*Med. Times.*

